# The physiology of intraoperative error: using electrokardiograms to understand operator performance during robot-assisted surgery simulations

**DOI:** 10.1007/s00464-023-09957-0

**Published:** 2023-03-02

**Authors:** Christopher D’Ambrosia, Eliah Aronoff-Spencer, Estella Y. Huang, Nicole H. Goldhaber, Garth R. Jacobsen, Bryan Sandler, Santiago Horgan, Lawrence G. Appelbaum, Henrik Christensen, Ryan C. Broderick

**Affiliations:** 1 Department of Computer Science and Engineering, University of California San Diego, La Jolla, CA USA; 2 College of Physicians and Surgeons, Columbia University, New York, NY USA; 3 Department of Medicine, University of California San Diego, La Jolla, CA USA; 4 Division of Minimally Invasive Surgery, Department of Surgery, University of California San Diego, La Jolla, CA USA; 5 Department of Psychiatry, University of California San Diego, La Jolla, CA USA; 6 Cognitive Robotics Laboratory, Department of Computer Science and Engineering, University of California San Diego, 9500 Gilman Drive, Mail Code 0404, La Jolla, CA USA

**Keywords:** Robotic surgery, Laparoscopy, Minimally invasive surgery, Surgical performance, Surgical education

## Abstract

**Background:**

No platform for objective, synchronous and on-line evaluation of both intraoperative error and surgeon physiology yet exists. Electrokardiogram (EKG) metrics have been associated with cognitive and affective features that are known to impact surgical performance but have not yet been analyzed in conjunction with real-time error signals using objective, real-time methods.

**Methods:**

EKGs and operating console point-of-views (POVs) for fifteen general surgery residents and five non-medically trained participants were captured during three simulated robotic-assisted surgery (RAS) procedures. Time and frequency-domain EKG statistics were extracted from recorded EKGs. Intraoperative errors were detected from operating console POV videos. EKG statistics were synchronized with intraoperative error signals.

**Results:**

Relative to personalized baselines, IBI, SDNN and RMSSD decreased 0.15% (S.E. 3.603e−04; *P* = 3.25e−05), 3.08% (S.E. 1.603e−03; *P* < 2e−16) and 1.19% (S.E. 2.631e−03; *P* = 5.66e−06), respectively, during error. Relative LF RMS power decreased 1.44% (S.E. 2.337e−03; *P* = 8.38e−10), and relative HF RMS power increased 5.51% (S.E. 1.945e−03; *P* < 2e−16).

**Conclusions:**

Use of a novel, on-line biometric and operating room data capture and analysis platform enabled detection of distinct operator physiological changes during intraoperative errors. Monitoring operator EKG metrics during surgery may help improve patient outcomes through real-time assessments of intraoperative surgical proficiency and perceived difficulty as well as inform personalized surgical skills development.

**Graphical abstract:**

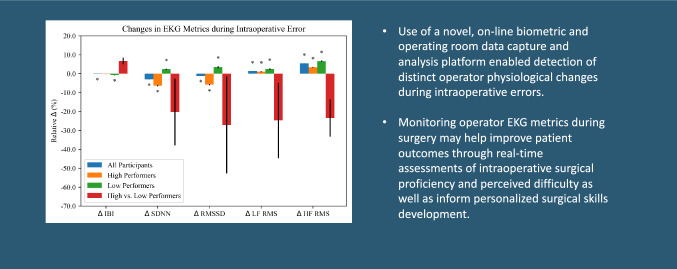

**Supplementary Information:**

The online version contains supplementary material available at 10.1007/s00464-023-09957-0.

Understanding the relationship between operator physiology and performance during robot-assisted surgery (RAS) has the potential to improve operating outcomes, surgical education, training, and skills assessment [1–3]. Time and frequency-domain electrokardiogram (EKG) metrics provide high-temporal resolution measures of surgeon physiology that are robust to sensing challenges in dynamic operating room (OR) environments [4]. These metrics have been associated with cognitive and affective features such as mental workload, acute stress, and cognitive fatigue that are known to impact surgical performance [5–7]. To explore the relationships between physiology and performance, subjective, manual ratings of surgical performance or error are then post-processed and correlated with physiological data and their implied cognitive or affective features [8–10]. Developing a more robust relationship between intraoperative EKG metrics and surgical performance, however, will require rater-independent, objective error annotation, high-temporal resolution error identification, and precise alignment of error markers with physiological signals. The present study seeks to develop on-line, automated detection and alignment of intraoperative errors with EKG signals to identify relationships between EKG statistics and surgical performance in real-time. Results suggest that operator EKG statistics change significantly during intraoperative errors and the magnitude of these changes varies with operator skill level.

Prior works examining EKG statistics in the context of surgical performance typically consist of retrospective analyses that often aggregate physiological metrics over the entire surgical procedure and use manual rating schemes to characterize per-procedure surgical performance. For example, Langelotz et al. associated perceived stress levels in surgeons during a 24-h shift with heart rate variability (HRV) metrics [11] while Bohm et al. examined HRV to measure mental strain in surgeons during laparoscopic surgery [7]. Joseph et al. used subjective measures of cognitive load to examine physiological differences in novice vs. expert surgeons during emergency surgery [12]. Wetzl et al. measured HRV and salivary cortisol levels to determine whether physiological indicators of stress were predictive of surgical performance during simulated OR crises [13]. Amirian et al. examined the correlations between HRV and laparoscopic simulation performance [14]. All of these studies use aggregated physiological statistics and subjective performance outcomes. Higher temporal resolution experiments examining intraoperative, rather than per-procedure performance and physiology, have been published but are largely reliant on retrospective, rather than on-line or real-time, analysis due to the need for manual annotation of surgical performance by expert surgeons [15].

The aim of this study is to analyze intraoperative time and frequency domain EKG statistics of operators participating in RAS simulation using automated annotation of surgical videos for performance metrics. Changes in EKG metrics are compared relative to personalized baselines during periods of operator error to those captured during periods of no error. These differences are then analyzed to examine the differences in EKG statistics for high and low-performing operators during error. It is hypothesized that changes in EKG metrics relative to baseline will be significantly different during error compared to no error and that changes in relative EKG metrics during errors would differ across levels of performance.

## Materials and methods

### Participants

Twenty participants were recruited for this study under institutional IRB approval. Of these participants, 5 were non-medical graduate students and the remainder were general surgery residents at various levels of training (Table 1). All participants were naïve to the hypotheses of the study prior to participation.

**Table 1:** Participant demographics

	All	GS	PGY-1	PGY-2	PGY-3	PGY-4
No. (%)	20 (100)	5 (25)	4 (20)	4 (20)	5 (25)	2 (10)
Sex						
Male (%)	14 (70)	5 (100)	2 (50)	2 (50)	3 (60)	2 (100)
Female (%)	6 (30)	0 (0)	2 (50)	2 (50)	2 (40)	0 (0)
Age (years)						
Median	28.5	27.0	26.0	30.5	30.0	32.0
IQR	26.8–31.0	26.0–28.0	26.0–26.5	29.5–32.5	29.0–30.0	31.5–32.5

GS—Non-medical graduate student

*PGY*-1 first year general surgery resident, *PGY*-2 s year general surgery resident, *PGY*-3 third year general surgery resident, *PGY*-4 fourth year general surgery resident, *IQR* Interquartile range from first quartile to third quartile

### Surgical system, simulation software and sensors

The da Vinci Xi robotic system console (Intuitive Surgical Ltd.) with the da Vinci Skills Simulator (DVSS) software (Surgical Science Sweden AB) was used for this study. The operator’s point-of-view (POV) video from the DVSS was recorded using a video capture card with a recording rate of 30 frames-per-second (fps). For EKG recording, we used a Polar H10 (Polar Inc.) chest-strap monitor with a sampling rate of 130 Hertz (Hz).

### Protocol

Each participant completed three simulation tasks during a single sitting without breaks. These tasks were completed in the same order for all participants. The tasks utilized were “Ring Rollercoaster 1,” “Ring Rollercoaster 3,” and “Wrist Articulation 1” in this order for all participants.

A Polar EKG monitor was fitted on each participant prior to beginning the first task and checked for appropriate positioning and data transfer. Four minutes of baseline EKG readings were then recorded as each participant sat still at the operating console. Following baseline recording, each participant was directed to start the first surgical simulation task and given no other instruction. After completion of the first task, each participant was directed to start the second simulation, also without additional instruction or feedback. After completion of the second simulation, each participant was directed to start the third and final simulation, also without additional instruction or feedback. Following completion of the final simulation, each participant was instructed to complete a demographics survey after which the EKG monitor was removed and the system was reset for future participants.

### Data capture, feature extraction, and synchronization

Both the video data stream and the EKG data stream were recorded on the same external computer. Operator POV videos were captured at a frame rate of 30 fps. Each frame was programmatically annotated for the presence or absence of error using pixel-based frequency and intensity filters, Hough transforms, and canny edge detection. Frame-by-frame error annotation provided 30 Hz resolution for intraoperative error.

EKG data was captured at a sampling rate of 130 Hz. Interbeat interval (IBI), standard deviation of N–N interval (SDNN), root-mean-square successive differences in N–N interval (RMSSD), low frequency (LF) signal (0.04–0.15 Hz), and high frequency (HF) signal (0.15–0.40 Hz) features were calculated from raw EKG data [16]. SDNN and RMSSD were calculated using a 30 s (s) sliding window [17] with a single sample stride resulting in trailing 30 s SDNN and RMSSD measures at 130 Hz resolution.

LF and HF signal components were isolated from raw data using Butterworth bandpass filters with cascaded second-order sections and passbands of (0.04–0.15 Hz) and (0.15–0.40 Hz), respectively. LF and HF signal powers were calculated using root-mean-square (RMS) amplitude.

Data streams were synchronized using Lab Streaming Layer [18]. For simulation measures, IBI, RMSSD and SDNN metrics were associated with the time-matched video frame from the operator POV. Each video frame’s duration (0.033 s) was used as a non-overlapping window for LF and HF RMS power. Baseline IBI, RMSSD, and SDNN metrics were averaged over the entire 4-min baseline recording. Baseline LF and HF RMS power were computed using the average RMS power across non-overlapping 0.033 s windows.

### Measures

#### Time-domain EKG statistics

**Fig. 1:**
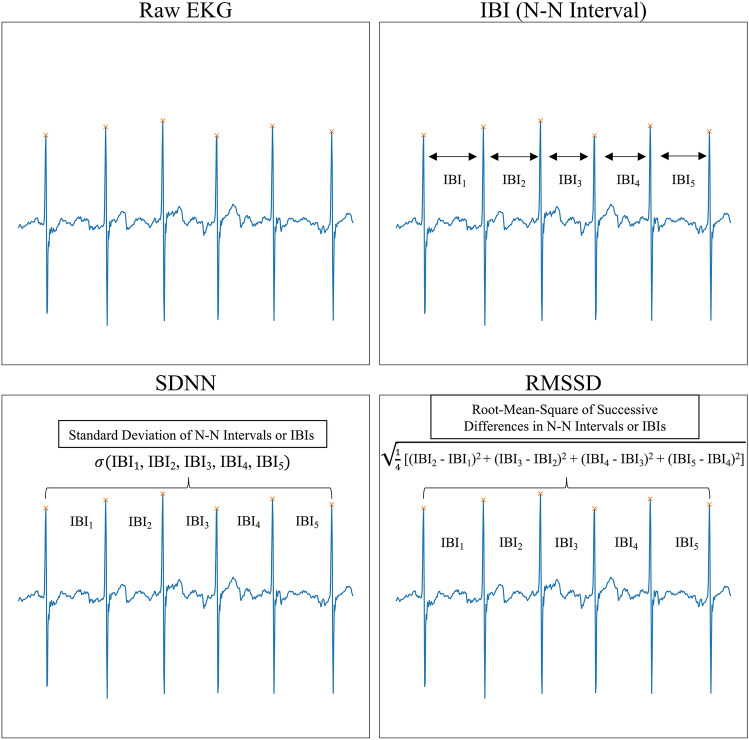
Time-Domain EKG Statistics. Interbeat interval (IBI) measures the time (ms) between two heart beats as indicated by the N–N interval. Standard deviation of N–N intervals (SDNN) measures the standard deviation of N–N intervals as measured by IBIs over a specified window of time. RMSSD measures the root mean square of successive differences in N–N intervals as measured by successive differences in IBIs over a window of time

IBI reflects the amount of time between successive heart beats (Fig. 1). A decrease in IBI is equivalent to an increase in heart rate which is a marker of increased sympathetic autonomic nervous system (SANS) activity or decreased parasympathetic autonomic nervous system (PANS) activity [19, 20]. Increased SANS activity has been associated with increased stress, mental and physical workload, and suppression of negative emotions [21].

Increases in SDNN are associated with increased vagal tone and therefore increased PANS activity [22]. Vagal tone and increased PANS activity has been correlated with resilience to mental, emotional, and physical stress as well as lower cognitive workload [23–25]. Increases in RMSSD are also associated with higher vagal tone and PANS activity [26].

#### Frequency-domain EKG statistics

**Fig. 2:**
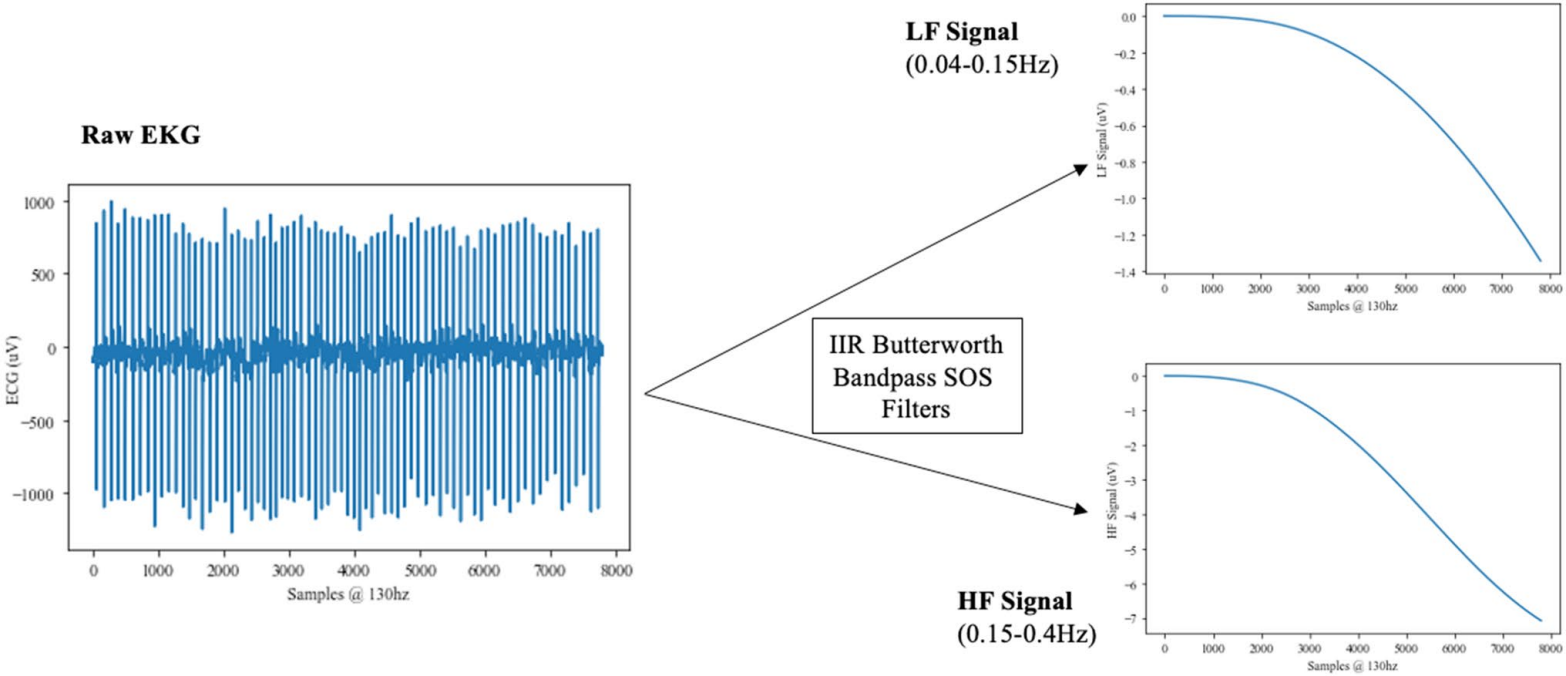
Time and frequency-domain EKG statistics. After acquiring a raw EKG signal, the raw signal is passed through a low-frequency bandpass filter (0.04–0.15 Hz) to obtain the LF component of the raw EKG signal. To obtain the HF component of the raw EKG signal, the raw signal is passed through a high-frequency bandpass filter (0.15–0.4 Hz). The RMS amplitudes of the LF and HF components are used to estimate the power of each component

LF power (Fig. 2) is an indicator of mixed SANS and PANS activity as well as baroreflex activity whereas HF power reflects vagal tone [26]. Increases in LF are associated with higher mental workload [27], and decreases in HF are associated with stress, panic, anxiety, or worry [28].

### Statistical analyses

#### All participants

EKG metrics relative to individual baselines were associated with intraoperative errors using separate linear mixed effects models for each metric. Intraoperative errors were modeled as a fixed effect and individual participant variance as a random effect. Identically structured models were used for each relative frequency-domain metric (LF RMS power, HF RMS power).

#### High and low performers

All participants were assigned to either a high, middle or low performance group based on the sum total of their simulation scores for all three tasks (Table 2). Simulation scores for each task are generated by the DVSS based on aggregated simulation statistics including time to completion, instrument distance traveled, instrument errors, and manipulation errors. The high performance group included the top-third (7 participants) by score. The low performance group included the bottom-third (7 participants) by score. The remainder of the participants were assigned to the middle performance group.

**Table 2:** Group demographics

	High performers	Middle performers	Low performers
No. (% of total)	7 (35)	6 (30)	7 (35)
Sex			
Male (%)	4 (57)	6 (100)	4 (57)
Female (%)	3 (43)	0 (0)	3 (43)
Age (years)			
Median (IQR)	28.0 (28.0–31.0)	29.5 (26.8–32.3)	28.0 (26.5–30.0)
Level of training			
GS	1 (14)	2 (33)	2 (29)
PGY-1	1 (14)	1 (17)	2 (29)
PGY-2	2 (29)	0	2 (29)
PGY-3	2 (29)	2 (33)	1 (14)
PGY-4	1 (14)	1 (17)	0 (0)
Total score			
Average	203.6	82.0	26.3
Median (IQR)	217.0 (146.5–256.5)	79.5 (69.8–89.3)	21.0 (11.0–41.0)
Time to completion (s)			
Average	582.6	789.5	1069.4
Median (IQR)	601.1 (396.1–737.5)	793.8 (730.2–825.3)	1036.3 (962.9–1181.8)
Instrument distance traveled (cm)			
Average	805.0	1199.6	1392.9
Median (IQR)	812.1 (626.3–874.6)	1235.7 (1088.1–1341.4)	1389.1 (1274.0–1523.2)
Intraoperative error (%)			
Average	15.7	26.5	10.9
Median (IQR)	16.9 (10.7–19.8)	28.9 (22.7–31.0)	10.8 (9.5–11.5)

Total score represents the sum total of all scores for three simulation tasks as calculated by the DVSS. Instrument distance traveled is the total distance that the end effectors of both robotic arms traveled for all three simulations. Time to completion is the total amount of time taken to complete all three simulation tasks. Intraoperative error is the percentage of video frames in which the participant is making an error relative to the total number of video frames required for that participant to complete all three simulations

High Performer and Low Performer groups were analyzed separately. For each group, the association between relative time-domain (IBI, SDNN, RMSSD) EKG metrics and intraoperative error were modeled using separate linear mixed effects models for each time-domain metric. Intraoperative error was modeled as a fixed effect and individual participant variance as a random effect. Identically structured models were used for each relative frequency-domain metric (LF RMS power, HF RMS power).

#### High vs. low performers

To compare High Performer and Low Performer group physiology during intraoperative error, separate linear mixed effects models were used for all relative time and frequency-domain metrics. Group membership was modeled as a fixed effect using Low Performer as reference level, and we modeled participant level variance as a random effect.

### Multiple comparison correction

We independently tested all 5 EKG metrics (IBI, SDNN, RMSSD, LF RMS, HF RMS) for all 4 groupings (all participants, high performers, low performers, high vs. low performers). Our uncorrected significance level was *P* = 0.01. Given a total of 20 independent tests in this study, we conservatively corrected our significance level by a factor of 20 to *P* = 0.0005.

## Results

### All participants

**Fig. 3:**
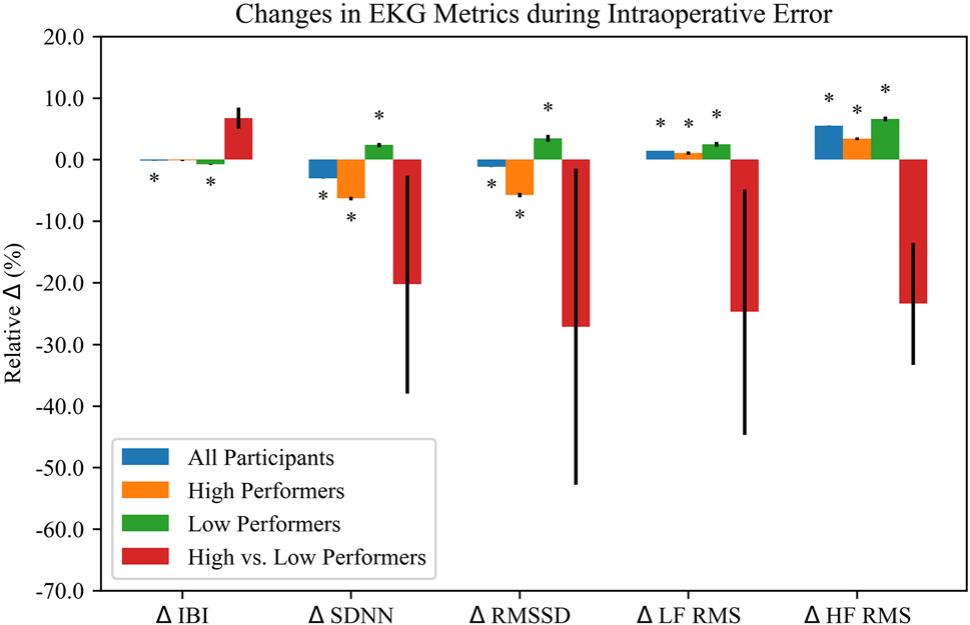
Results for all analyses across all EKG metrics. *P < 0.0005

Relative IBI for all participants decreased 0.15% (Standard Error: 3.60e−04; *P* = 3.25e−05) during error as compared to no error. Relative SDNN decreased 3.08% (S.E.: 1.60e−03; *P* < 2e−16), and relative RMSSD decreased 1.19% (S.E.: 2.63e−03; *P* = 5.66e−06) during error as compared to no error. Relative LF RMS power increased 1.44% (S.E.: 2.34e−03; *P* = 8.38e−10), and relative HF RMS power increased 5.51% (S.E.: 1.95e−03; *P* = 2e−16) during error as compared to no error (Table 3 and Fig. 3).

**Table 3:** Changes in EKG metrics during error

Group/Metric	Estimate	Standard error (SE)	*P*
All participants			
IBI	− 1.497e−03	3.603e−04	3.25e−05
SDNN	− 3.078e−02	1.603e−03	< 2e−16
RMSSD	− 1.194e−02	2.631e−03	5.66e−06
LF RMS power	1.435e−02	2.337e−03	8.38e−10
HF RMS power	5.509e−02	1.945e−03	< 2e−16
High performers			
IBI	− 1.310e−03	7.635e−04	0.0861
SDNN	− 6.318e−02	2.846e−03	< 2e−16
RMSSD	− 5.773e−02	3.683e−03	< 2e−16
LF RMS power	1.073e−02	2.914e−03	0.000230
HF RMS power	3.393e−02	2.241e−03	< 2e−16
Low performers			
IBI	− 7.773e−03	6.027e−04	< 2e−16
SDNN	2.370e−02	3.284e−03	5.28e−13
RMSSD	3.460e−02	5.570e−03	5.24e−10
LF RMS power	2.499e−02	3.918e−03	1.81e−10
HF RMS power	6.608e−02	3.975e−03	< 2e−16
High vs. low performers			
IBI	0.06730	0.01742	0.00226
SDNN	− 0.2027	0.1767	0.2740
RMSSD	− 0.2709	0.2566	0.3120
LF RMS power	− 0.2474	0.1992	0.238
HF RMS power	− 0.23411	0.09878	0.0354

All participants includes all participants in the study with changes in relative EKG metrics for error as compared to no error. High Performers includes the top-7 performers by sum total DVSS score across all three simulations. Low Performers includes the bottom-7 performers by sum total DVSS score across all three simulations. High vs. Low Performers indicates a comparison of changes in relative EKG metrics during error for High Performers and Low Performers. Estimate represents the fixed effect coefficient

### High and low performers

In the High Performer group, relative SDNN and relative RMSSD decreased 6.3% (S.E.: 2.846e−03, *P* < 2e−16) and 5.8% (S.E.: 3.683e−03, *P* < 2e−16), respectively, during error as compared to no error while relative IBI showed no significant change. Relative LF and HF RMS powers increased 1.1% (S.E.: 2.914e−03, *P* = 0.000230) and 3.4% (S.E.: 2.241e−03, *P* < 2e−16), respectively, during error as compared to no error (Table 3 and Fig. 3).

In the Low Performer group, relative IBI decreased 0.8% (S.E.: 6.027e−04, *P* < 2e−16) during error as compared to no error while relative SDNN and relative RMSSD increased 2.4% (S.E.: 3.284e−03, *P* = 5.28e−13) and 3.5% (S.E.: 5.570e−03, *P* = 5.24e−10), respectively. Relative LF and HF RMS powers increased 2.6% (S.E.: 3.918e−03, *P* = 1.81e−10) and 6.6% (S.E.: 3.975e−03, *P* ≤ 2e−16), respectively, during error as compared to no error (Table 3 and Fig. 3).

### High vs. low performers

Differences in changes in all EKG metrics during error for high performers as compared to low performers did not meet our corrected significance threshold of *P* = 0.0005 (Table 3 and Fig. 3).

## Discussion

Potential cognitive and affective determinants of surgical performance, including stress, mental workload, and valence, have been explored in prior research [29–31]. These determinants have been associated with both physiological and neurophysiological metrics including EKG statistics [32, 33]. Time and frequency-domain EKG measures are particularly relevant given their proposed mappings to cognitive and affective features [34]. Additionally, these EKG measures have been associated with surgical performance independent of any cognitive or affective mediators [30]. Surgical education and surgical performance improvement are of paramount importance to patients, surgeons, and surgical trainees. EKG-based biometrics coupled with objective intraoperative error detection offer a potential method for personalized surgical performance evaluation and targeted surgical skill improvement. Precision surgical skill evaluation and development may drive improved patient outcomes through higher operator technical proficiency. Prior work, however, neglects the potential of on-line EKG analysis coupled with automated surgical error detection to capture high temporal-resolution indicators of intraoperative success or failure.

In this study, we built and deployed a novel, on-line operating room data capture and analysis platform to test the hypotheses that relative time and frequency-domain EKG metrics of operators performing RAS would change significantly during intraoperative error. Our results only consider detected errors and the EKG statistics associated with those detected errors. While other factors, such as stress, may also influence the physiological correlates of error, stress can occur without error and error can occur without stress. Our analysis does not distinguish between the potential effects of error on stress or the potential effects of stress on error, and our results suggest that all relative time-domain metrics (IBI, SDNN, RMSSD) and all relative frequency-domain metrics (LF and HF RMS power) demonstrate significant changes during error as compared to no error. The direction of these changes largely aligns with proposed associations between EKG statistics and cognitive or affective determinants of surgical performance. Decreases in relative IBI, SDNN, and RMSSD, and increases in relative LF RMS power during intraoperative error may reflect an increase in operator stress and mental workload as well as a more negative affective state. The increase in relative HF RMS power during intraoperative error, however, may suggest a decrease in stress, panic, anxiety or worry [35].

For high performers, decreases in relative SDNN and RMSSD associated with intraoperative error are possibly reflective of greater stress, mental workload and negative valence. However, increases in relative LF RMS power, indicative of higher stress and workload, were of smaller magnitude than increases in relative HF RMS power. HF RMS power is reported to be indicative of lower anxiety or worry [35]. For Low Performers, only relative IBI decreased during error while all other metrics increased. For both groups, the direction of changes in relative IBI, LF RMS power and HF RMS power were similar, potentially indicating common physiological associations with intraoperative error regardless of performance status. However, the magnitude of these changes was not identical.

In the previous group analyses, EKG statistics during error were compared to those during no error, and the differences between error and no error were of sufficient magnitude to be detected. Examination of the differences in relative IBI and HF RMS *during* error for high as compared to would benefit from further investigation in a higher-powered group comparison. Given the relatively small magnitude of differences in the error compared to no error analyses, detecting even smaller magnitude differences in *error compared to error* across two groups would benefit from a larger cohort size.

There are several limitations of this study. First, the number of participants was neither large nor evenly distributed across skill level, potentially skewing cohort analysis. Second, the use of trailing 30 s windows for SDNN and RMSSD statistics implies that the metrics associated with a particular time point reflect cardiac activity for the entire 30 s up to and including that time point. Third, using bandpass filters and RMS power calculations for frame-by-frame LF and HF power measurements are accompanied by a tradeoff in the settling time of the filter. Fourth, we do not exclude individuals on chronotropic or inotropic medications nor do we correct for the effect of these medications. While this may impact our results, all reported EKG statistics are analyzed relative to each individual participant's personalized baseline EKG. As these baseline characteristics are captured immediately prior to the simulation tasks, the effects of medication are likely to be consistent across both baseline and simulation EKG recording periods. Finally, these results may be affected by other variables such as movement at the console and time at the console. Based on our observations, low performers tended to move and shift body positions more frequently than high performers, potentially indicative of discomfort at the console. Low performers also required more time to complete the simulation tasks.

Despite these limitations, this study shows that a novel, on-line operating room data capture and analysis platform can enable the detection of distinct physiological changes associated with intraoperative error. These changes are largely consistent with known relationships between EKG metrics and cognitive or affective factors that impact performance [36]. While this preliminary study only proposes potential biomarkers of intraoperative error, these biometric error signals have the potential to improve our understanding of surgical proficiency and surgical training. Using EKG-based metrics of error, challenge, or difficulty could help identify intraoperative tasks and procedures that pose significant challenges to operators and guide personalized training on an individual-by-individual basis. Coupling these EKG-based metrics with other markers of error, challenge or difficulty such as electroencephalogram (EEG) statistics, eye-tracking metrics, and galvanic skin response could provide a multidimensional approach to understanding, predicting, and improving surgical performance. This data highlights the possibility of using objective biometric data capture and error detection to monitor intraoperative surgical proficiency and perceived intraoperative difficulty in order to improve patient outcomes as well as potentially guide more precise, personalized RAS training curriculums. Future studies may focus on use of real-time error detection and physiological analysis to reduce error commission and mitigate negative cognitive or affective states through coaching or other skills development tools in an individualized surgical learning curriculum.

## Supplementary Information

Below is the link to the electronic supplementary material.

Supplementary file1 (MP4 17463 KB)
